# Transplant Trial Watch

**DOI:** 10.3389/ti.2024.13593

**Published:** 2024-08-26

**Authors:** Simon R. Knight, John Fallon, Reshma Rana Magar

**Affiliations:** ^1^ Centre for Evidence in Transplantation, Nuffield Department of Surgical Sciences, University of Oxford, Oxford, United Kingdom; ^2^ Oxford Transplant Centre, Churchill Hospital, Oxford, United Kingdom

**Keywords:** systematic review/meta-analysis, kidney transplantation (KT), delayed graft function, randomised controlled trial, antibody mediated rejection (ABMR)

## Aims

This study aimed to evaluate whether low-chloride solutions would reduce the incidence of delayed graft function and improve acid-base and electrolyte balance in kidney transplant recipients.

## Interventions

Three electronic databases, including MEDLINE, EMBASE, and Cochrane, were searched for relevant literature. Studies were screened and data were extracted by two independent reviewers. The Cochrane Risk of Bias Tool for Randomized Trials 2 (RoB2) was used to assess the quality of the included randomised controlled trials.

## Participants

12 studies were included in the review.

## Outcomes

The primary outcome was the incidence of delayed graft function. The secondary outcomes included end of surgery chloride, bicarbonate, pH, base excess (BE) and potassium, and post-operative creatinine and urine output.

## Follow-Up

N/A.

## CET Conclusions

*by Reshma Rana Magar*

This systematic review aimed to examine whether using balanced crystalloid solutions would result in better clinical outcomes in kidney transplant recipients, compared to normal saline. Twelve studies were included, all of which were randomised controlled studies. Study selection, data extraction and quality assessment were performed in duplicate. The meta-analyses revealed that the use of balanced low-chloride solutions resulted in a significant reduction in the incidence of delayed graft function (DGF), and improved acid-base and electrolyte control in kidney transplant patients, leading the authors to conclude that balanced lower-chloride solutions can be used as a safe alternative to normal saline and may even lead to better post-transplant outcomes. It is important to note that while the difference in the occurrence of delayed graft function was significant in the overall analysis that included both living and deceased donors, the subgroup analysis showed that this difference was only significant for deceased donor transplant recipients and not for living donor transplant recipients. A potential reason for this could be that only a few studies (three studies) reported DGF outcomes for living-donor transplant recipients, out of which one study had zero events for both arms. Heterogeneity was negligible for most of the primary outcomes. However, the influence of potential confounders were not accounted for in the analyses.

## Trial Registration

PROSPERO - CRD42023447301.

## Funding Source

No funding received.

## Aims

They aim to assess the safety of CD38 monoclonal antibody therapy, felzartamab in the treatment of AMBR in kidney transplantation.

## Interventions

Participants received either felzartamab (9 IV doses of 16 mg over 20 weeks) or placebo.

## Participants

22 adult kidney recipients with AMBR at least 180 days after transplantation and a eGFR >20 mL/min/1.73 m^2^.

## Outcomes

Primary outcome was ther safety and side effect profiles of felzartamab. Secondary outcomes included: resolution of ABMR, level of microvascular inflammation, classifier score of ABMR, DSA assessment, NK-cell count, donor cfDNA & eGFR slope.

## Follow-Up

52 Weeks.

## CET Conclusions

*by John Fallon*

The investigators present a blinded, placebo-controlled RCT for the safety of potentially exciting therapy for ABMR, felzartamab. They find an effective early response during the treatment window of the first 24 weeks, with resolution to chronic (inactive) rejection or no rejection in 9 of the 11 (82%) who received the anti-CD38, with only 2 of 10 (20%) having resolution in the placebo group. This was accompanied by reduction in the microvascular inflammation scores for those who received felzartamab compared to placebo. In the 6-month observation period following treatment the differences between the groups begins to wane, with 3 of those who had inactivity on biopsy at 6 months having activity at 12 months. Within the placebo group there is still only 2 of 10 with no activity on biopsy at 12 months, but these are 2 different participants from those at 6 months, who have become active. Along with this, the differences in microvascular injury score and probability score for AMBR have become narrower. The relevant clinical manifestation of this was the 1-year eGFR slope was shallower with felzartamab at −0.39 mL/min/1.73 m^2^, compared with −4.53 mL/min/1.73 m^2^ in placebo. It appears likely that during the treatment period there is an effect of the anti-CD38 on activity, but that without regular dosing, or additional treatments titrated to biopsy results this effect diminishes over time. With NK-cell depletion, the key safety considerations is infections, which were unsurprisingly numerically higher, but not significant in the felzartamab group, 91% compared with 64% in the control. The inherent limitation of small sample size within this safety RCT means commenting on efficacy or the risk benefit with adverse infection is not possible, but they have performed a robustly designed study demonstrating safety of felzartamab with convincing preliminary evidence for a larger multi-centre/multi-national efficacy study for the treatment for a condition which to date has no approved therapies.

### Jadad Score

5.

### Data Analysis

Strict intention-to-treat analysis.

### Allocation Concealment

Yes.

### Trial Registration

ClinicalTrials.gov - NCT05021484; EudraCT-2021-000545-40.

### Funding Source

Industry funded.

## Clinical Impact Summary

*by Simon Knight*

Management of antibody mediated rejection (ABMR) in renal transplant recipients remains a significant challenge. Antibody removal with a combination of plasma exchange, steroid and intravenous immunoglobulin remains standard of care, with no other therapies recommended in consensus guidelines [1]. Randomised trials of agents targeting plasma cells such as rituximab and bortezomib have failed to show convincing clinical benefit [2, 3]. The anti-IL6 antibody Clazakizumab showed promising results in phase 2 studies, although a recent phase 3 study was terminated early due to lack of efficacy [4, 5]. Clinical studies in this area are challenging due to difficulties in identifying patients and relatively slow recruitment rates.

In a recent issue of the New England Journal of Medicine, Mayer and colleagues report the results of a phase 2 trial of the CD38 monoclonal antibody felzartamab in renal transplant recipients with late antibody mediated rejection [6]. This small safety study is well designed, with block randomisation and placebo control to ensure blinding and allocation concealment. The investigators randomised 22 patients with late ABMR to 9 infusions of felzartamab over 20 weeks, or placebo infusions. Patients were then followed for a further 6 months following completion of treatment.

The primary focus of the study was safety. Eight patients had mild to moderate infusion reactions with felzartamab, but there were very few serious adverse events and these did not differ significantly between groups. Infections were numerically but not significantly higher in the treatment arm.

Interesting efficacy signals were also seen. At the end of treatment, there was resolution of ABMR in 82% of treated patients compared to 20% of controls. Microvascular inflammation, molecular risk of rejection score and cell-free DNA were all lower in the treatment arm. However, in the 6 months following cessation of treatment, 3 of 9 responding patients showed recurrence with increase in molecular and biomarker activity.

These results are very promising for treatment of a challenging condition. Strong conclusions are limited by sample size and a very narrow patient population, but they do suggest that felzartamab may have a role to play in the management of ABMR. The recurrences seen after the end of treatment suggest that careful monitoring and further dosing may be required for some patients.

### Clinical Impact

4/5.
